# The relationship between serum levels of miR-342 and miR-148a and acute lung injury in sepsis patients

**DOI:** 10.1515/med-2025-1343

**Published:** 2026-01-28

**Authors:** Meirong Shen, Tingting Cai, Jianlong Zhu, Zuan Yin

**Affiliations:** Department of Critical Care Medicine, Ganzhou People’s Hospital, Ganzhou City, Jiangxi Province, China; Department of Infections Diseases, Ganzhou People’s Hospital, Ganzhou City, Jiangxi Province, China; Department of Emergency, Jiangxi Provincial People’s Hospital, Nanchang City, Jiangxi Province, China

**Keywords:** sepsis, acute lung injury, micro RNA-342, micro RNA-148a, prediction

## Abstract

**Objectives:**

To investigate the relationship between serum miR-342, miR-148a, and acute lung injury (ALI) in sepsis patients.

**Methods:**

A total of 177 sepsis patients were divided into ALI and non-ALI groups; ALI patients were further classified as mild, moderate, or severe. 162 pneumonia patients served as controls. Serum miR-342 and miR-148a levels were measured using RT-qPCR.

**Results:**

Sepsis patients had lower miR-342 and higher miR-148a than controls (p<0.05). Compared with non-ALI patients, the ALI group showed higher miR-148a, PaCO2, APACHE II scores, and RI, but lower miR-342 and OI (p<0.05). miR-342 decreased and miR-148a increased with ALI severity. Correlation analysis revealed that miR-342 was negatively correlated with PaCO2, APACHE II, and RI, and positively with OI, while miR-148a showed opposite trends. The AUC for predicting ALI using miR-342, miR-148a, and their combination was 0.818, 0.775, and 0.896, respectively.

**Conclusions:**

Sepsis patients with ALI exhibit low serum miR-342 and high miR-148a, and their combination can effectively predict ALI occurrence.

## Introduction

Sepsis is a common complication following trauma, defined as a life-threatening organ dysfunction syndrome caused by a dysregulated host response to infection [1], 2]. The lungs are also the most commonly affected and vulnerable organs in sepsis, often presenting as acute respiratory distress syndrome (ARDS). Sepsis-associated ARDS increases patient mortality; therefore, early identification of acute lung injury is crucial [3]. Currently, several biomarkers have been identified for the early prediction of acute lung injury in sepsis patients, such as Z-DNA binding protein 1 (ZBP1) and ferroptosis-related markers including transforming growth factor-β1 (TGF-β), ubiquitin-specific peptidase 7, and Yes-associated protein [4], 5]. However, further research is needed to identify additional biomarkers to help improve predictive accuracy. MicroRNAs (miRNAs),as small non-coding RNAs, can inhibit protein translation or induce mRNA degradation by specifically recognizing the 3′ untranslated region of target gene mRNAs, thereby regulating gene expression at the post-transcriptional level. Thus, miRNAs can influence acute lung injury by regulating the expression of downstream inflammatory factors, and they have been identified as critical regulatory factors in the inflammatory response of acute lung injury [6]. The study by Qian et al. [7]demonstrated that civetiric acid can alleviate sepsis-induced acute lung injury by targeting and upregulating the expression of miR-744-5p, thereby inhibiting the inflammatory response. García-Concejo A et al. [8] found that extracellular vesicle-derived miR-342-3p, miR-148a-3p, and others can effectively distinguish postoperative septic shock from non-septic shock. Besides, Zhu et al. [9] demonstrated that miR-342 was downregulated in lipopolysaccharide-induced human adenocarcinomic human alveolar basal epithelial cells (A549 cells), accompanied by increased pro-inflammatory cytokines and cell apoptosis, suggesting a protective role of miR-342 in acute lung injury in mice. do Nascimento MF et al. [10] found that miR-148a and other miRNAs in circulating extracellular vesicles may serve as potential biomarkers and mediators of sepsis-induced acute respiratory distress syndrome. Ren et al.7 [11] found that miR-148a-3p was upregulated in patients with acute lung injury complications caused by severe COVID-19 pneumonia. Both miR-342 and miR-148a are associated with sepsis and acute lung injury, respectively. But their roles and clinical significance in septic patients with concurrent acute lung injury remain unclear. Therefore, this study aims to investigate the serum levels of miR-342 and miR-148a in septic patients and their relationship with the occurrence of acute lung injury, with the goal of providing valuable insights for clinical diagnosis and treatment.

## Materials and methods

### General information

A cohort of 177 septic patients, admitted to our hospital from June 2020 to April 2023, constituted the study group. The patients’ ages ranged from 45 to 87 years, with a mean age of (60.78 ± 10.36) years. Among them, 105 were male and 72 were female. According to the criteria for acute lung injury [12], the septic patients were divided into two groups: the acute lung injury group with 56 cases and the non-acute lung injury group with 121 cases. Patients with acute lung injury were further classified according to disease severity into the mild group [200<oxygenation index (OI)≤300 [13], n=21], the moderate group (100<OI≤200, n=25), and the severe group (OI≤100, n=10). A total of 162 patients with pneumonia were selected during the same period as the control group. Written informed consent was obtained from all participants or their legal representatives prior to enrollment.

**Figure 1:**
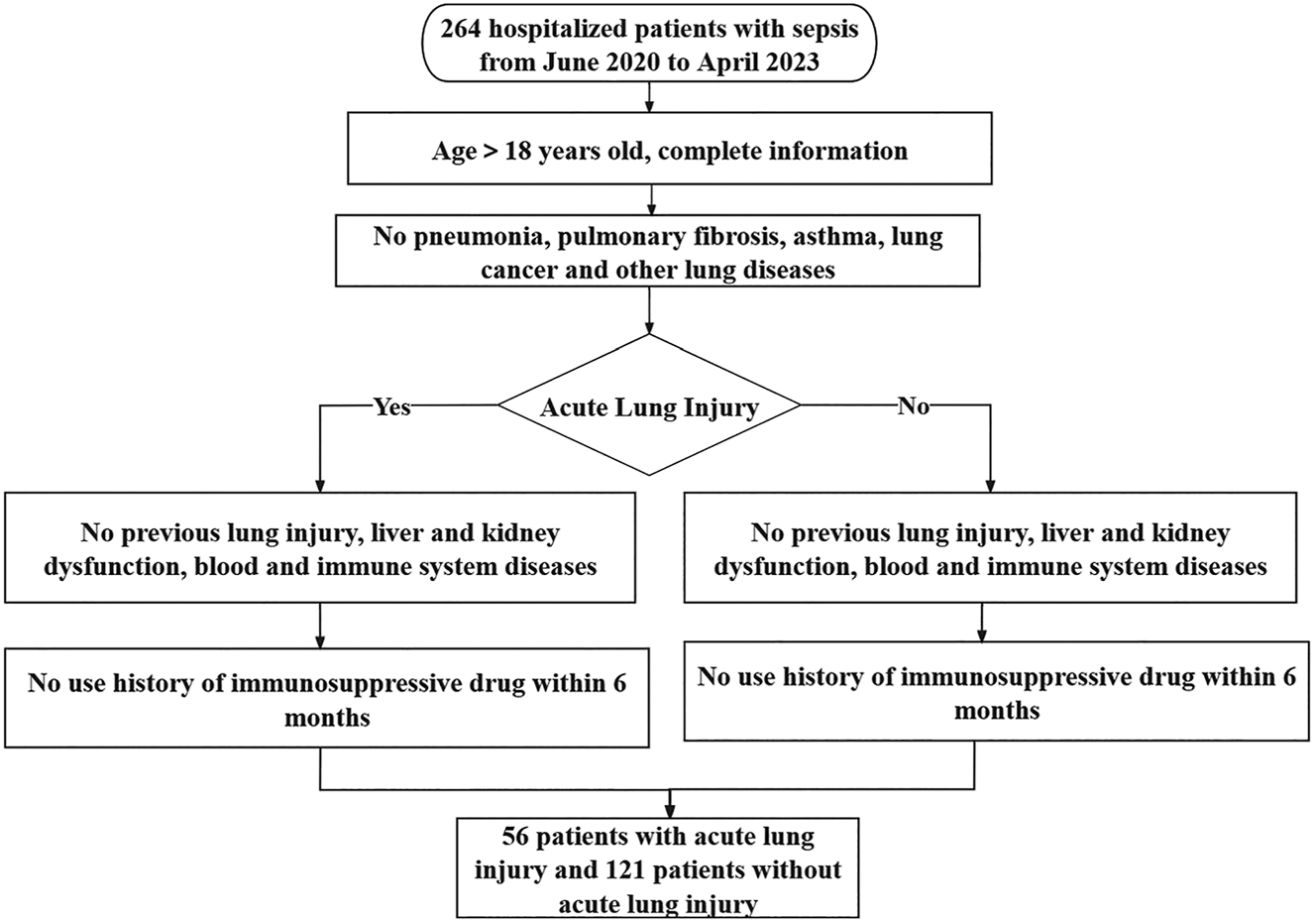
The flowchart of case collection.

**Figure 2:**
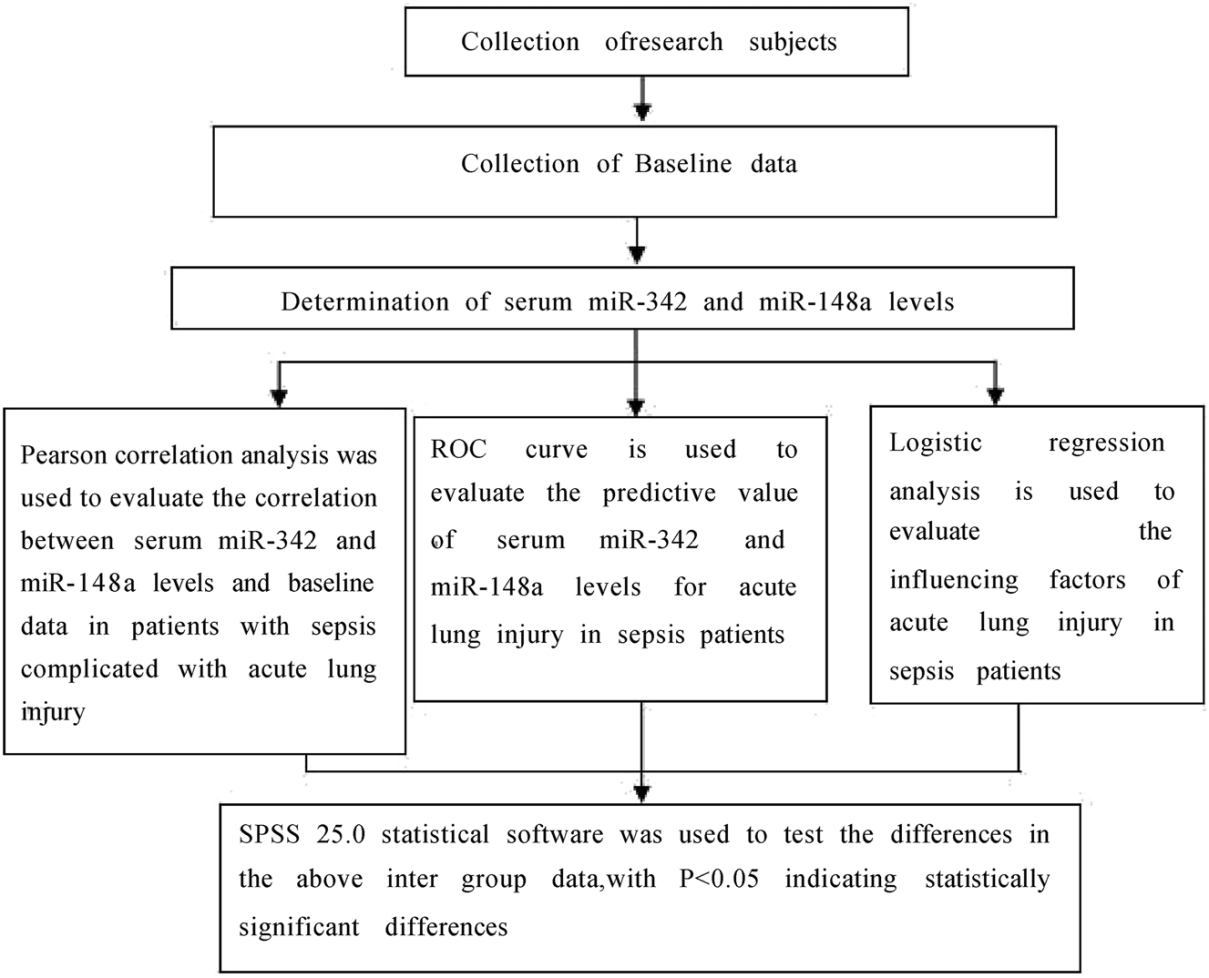
Research content and structural diagram.

Inclusion criteria: (1) Diagnosis of sepsis and acute lung injury according to the diagnostic criteria [12], 14]; (2) Age>18 years with complete baseline data; (3) No previous history of acute lung injury; (4) The presence of underlying diseases was limited to hypertension and/or diabetes. Exclusion criteria: (1) Patients with autoimmune diseases or hematological disorders; (2) Patients with pulmonary diseases such as pneumonia, pulmonary fibrosis, asthma, or lung cancer; (3) Patients with a history of immunosuppressive therapy in the past 6 months; (4) Patients with severe liver or kidney dysfunction. The patient selection and group allocation process is detailed in Figure 1. The research content and structural diagram are shown in Figure 2.

### Methods

#### Collection of baseline data

The gender, age, body mass index (BMI), smoking history, alcohol consumption history, primary site of infection, hypertension, diabetes, mechanical ventilation, oxygen therapy, arterial partial pressure of oxygen (PaO2), arterial partial pressure of carbon dioxide (PaCO2), procalcitonin (PCT), C-reactive protein (CRP), white blood cell count (WBC), forced expiratory volume in 1 s as a percentage of predicted value (FEV1), forced expiratory volume in 1 s to forced vital capacity ratio (FEV1/FVC), Acute Physiology and Chronic Health Evaluation II (APACHE II) score, and respiratory index (RI) of septic patients were collected.

#### Measurement of serum levels of miR-342 and miR-148a

Upon admission, prior to the initiation of specific sepsis treatment and before the clinical manifestation of acute lung injury, 3 mL of venous blood was collected from septic patients and placed in a dry centrifuge tube. After standing at room temperature for 30–60 min, the tube was centrifuged at 3,000 rpm (radius of 10 cm) at 4 °C for 15 min, and the upper serum was collected and stored at −80 °C. Total RNA was extracted from the thawed serum samples using Trizol reagent (Changzhou Beyotime Biotechnology Co., Ltd., Catalog No.: BYX1617P). Following verification of RNA integrity, purity, and concentration, cDNA was synthesized using a reverse transcription kit (Shanghai Yanhui Biotechnology Co., Ltd., Catalog No.: FSQ-301). Real-time quantitative PCR (RT-qPCR) was performed to analyze the gene expression of miR-342 and miR-148a using the prepared reaction system. The primer sequences are shown in Table 1. The reaction system consisted of 1 μL each of the upstream and downstream primers, 2 μL of cDNA (50 ng/μL), 10 μL of SYBR Green Realtime PCR Master Mix (Shanghai Huzhen Industrial Co., Ltd., Catalog No.: QPK-201), and ddH2O added to a final volume of 20 μL. The relative serum levels of miR-342 and miR-148a were calculated using the 2^−ΔΔCT^ method.

**Table 1:** Primer sequences for RT-qPCR.

Gene	Upstream primer 5′-3′	Downstream primer 5′-3′
miR-342	GTG​AGA​CAT​CAG​GTA​TCT​GCG​C	GAG​AGC​TAC​CGA​TCG​CTA​C
miR-148a	AGT​ATG​ATC​GCA​CGG​CCA​TC	GTA​TCA​CCG​TCC​CAG​GTG​TC
U6	GCA​CTC​TAC​TCG​ACG​GTC​AGA​G	CGT​GCC​AGA​CTG​TAT​CCG​AG

### Statistical analysis

Data analysis was performed using SPSS 25.0 software. Normally distributed continuous data, presented as mean ± standard deviation (SD), were compared between groups using independent sample *t*-tests, among three groups using one-way analysis of variance (ANOVA), and between pairs of groups using the Student-Newman-Keuls (SNK-q) test. Categorical data were presented as n (%) and compared using chi-square tests. Pearson correlation analysis was used to assess the relationships between serum miR-342 and miR-148a levels and key clinical parameters (e.g., PaCO2, APACHE II scores, RI, OI) across all septic patients. Receiver operating characteristic (ROC) curve analysis was employed to assess the predictive value of serum miR-342 and miR-148a levels for the occurrence of acute lung injury in septic patients. Logistic regression analysis was performed to identify factors associated with the development of acute lung injury in septic patients. A significance level of p<0.05 was considered statistically significant.

### Ethics approval

The studies involving human participants were reviewed and approved by the Ethics Committee of Ganzhou People’s Hospitaland (No.GZSRMYY2020040012) and with the 1964 Helsinki Declaration. Written informed consent to participate in this study was provided by the participants.

## Results

### Comparison of serum miR-342 and miR-148a levels between the control group and the study group

The serum miR-342 level in the study group was lower than that in the control group, while the miR-148a level was higher than that in the control group. The differences were statistically significant (p<0.05). See Table 2.

**Table 2:** Comparison of Serum miR-342 and miR-148a Levels Between the Control Group and the Study Group ( $\bar{x}$ ± *s*).

Groups	number(n)	miR-342/U6	miR-148a/U6
Control group	162	1.67 ± 0.32	0.64 ± 0.16
Study Group	177	0.94 ± 0.21	1.12 ± 0.23
*t*	–	25.029	22.112
p	–	<0.001	<0.001

### Comparison of baseline data between non-acute lung injury group and acute lung injury group

The non-ALI and ALI subgroups did not differ significantly regarding gender, age, BMI, smoking or alcohol consumption history, primary site of infection, comorbidities (hypertension, diabetes), use of mechanical ventilation or oxygen therapy, or levels of PaO_2_, PCT, CRP, WBC, FEV1, and FEV1/FVC (p>0.05). The acute lung injury group had significantly higher PaCO_2_, APACHE II scores, and RI, and lower OI compared to the non-acute lung injury group (p<0.05). See Table 3.

**Table 3:** Comparison of baseline data between non-acute lung injury group and acute lung injury group (( $\bar{x}$ ± *s*)/n(%)).

Items	No acute lung injury group (n=121)	Acute lung injury group (n=56)	t/χ^2^	p-Value
male [n (%)]	72 (59.50)	33 (58.93)	0.005	0.942
age (year)	60.74 ± 10.03	60.82 ± 10.56	0.049	0.961
BMI, kg/m^2^	22.85 ± 3.04	22.97 ± 2.96	0.246	0.806
Smoking history [n (%)]	68 (56.20)	31 (55.36)	0.011	0.917
Drinking history [n (%)]	19 (15.70)	9 (16.07)	0.004	0.950
Primary infection site [n (%)]			0.437	0.933
Lung	48 (39.67)	23 (41.07)		
Abdominal cavity	29 (23.97)	15 (26.79)		
Intracranial	20 (16.53)	9 (16.07)		
Urinary system	24 (19.83)	9 (16.07)		
hypertension [n (%)]	62 (51.24)	29 (51.79)	0.005	0.946
diabetes [n (%)]	26 (21.49)	12 (21.43)	0.000	0.993
Mechanical ventilation [n (%)]	22 (18.18)	12 (21.43)	0.260	0.610
Oxygen therapy [n (%)]	23 (19.01)	15 (26.79)	1.373	0.241
PaO_2_, mmHg	62.10 ± 9.49	60.35 ± 9.07	1.157	0.249
PaCO_2_, mmHg	40.62 ± 5.19	50.38 ± 6.13	10.974	<0.001
PCT, ng/mL	0.32 ± 0.10	0.33 ± 0.13	0.561	0.576
CRP, μg/mL	10.36 ± 3.17	10.48 ± 3.22	0.233	0.816
WBC( × 10^9^/L)	9.68 ± 2.16	9.71 ± 2.20	0.085	0.932
APACHE Ⅱ score (score)	12.32 ± 3.61	17.46 ± 5.02	7.746	<0.001
Lung injury score (score)	–	1.28 ± 0.40	–	–
FEV1, %	59.96 ± 12.35	61.77 ± 9.46	0.972	0.332
FEV1/FVC, %	64.90 ± 10.74	63.23 ± 9.81	0.988	0.324
OI	318.75 ± 69.84	226.30 ± 67.92	8.261	<0.001
RI	0.86 ± 0.15	1.62 ± 0.53	14.602	<0.001

### Comparison of serum levels of miR-342 and miR-148a between non-acute lung injury group and acute lung injury group

Patients in the ALI subgroup exhibited significantly lower serum levels of miR-342 and significantly higher levels of miR-148a compared to those in the non-ALI subgroup (p<0.05). See Table 4.

**Table 4:** Comparison of serum levels of miR-342 and miR-148a between non-acute lung injury group and acute lung injury group( $\bar{x}$ ± *s*).

Groups	number, n	miR-342/U6	miR-148a/U6
No acute lung injury group	121	1.04 ± 0.23	1.02 ± 0.22
Acute Lung injury group	56	0.73 ± 0.19	1.33 ± 0.25
t	–	8.790	8.345
P	–	0.000	0.000

### Comparison of serum miR-342 and miR-148a levels in patients with acute lung injury of different severity

The serum miR-342 levels in patients with mild, moderate, and severe acute lung injury decreased sequentially, while miR-148a levels increased accordingly. The differences among groups were statistically significant (p<0.05). See Table 5.

**Table 5:** Comparison of serum miR-342 and miR-148a levels in patients with acute lung injury of different severity( $\bar{x}$ ± *s*).

Groups	number, n	miR-342/U6	miR-148a/U6
Mild group	21	0.86 ± 0.19	1.16 ± 0.24
Moderate group	25	0.72 ± 0.18^a^	1.35 ± 0.25^a^
Severe group	10	0.48 ± 0.14^ab^	1.64 ± 0.31^ab^
*F*	–	15.533	11.889
p	–	0.000	0.000

^a^indicates p<0.05 compared with the mild group; ^b^indicates p<0.05 compared with the moderate group.

### Correlation between serum levels of miR-342 and miR-148a and baseline data in septic patients with acute lung injury

Pearson correlation analysis revealed that serum miR-342 levels in septic patients with acute lung injury were negatively correlated with PaCO_2_, APACHE II scores, and RI, and positively correlated with OI (p<0.05). On the other hand, miR-148a levels were positively correlated with PaCO_2_, APACHE II scores, and RI, and negatively correlated with OI (p<0.05). See Table 6.

**Table 6:** Correlation between serum levels of miR-342 and miR-148a and baseline data in septic patients with acute lung injury.

Items	miR-342		miR-148a	
	r	p-Value	r	p-Value
PaCO_2_	−0.519	<0.001	0.540	<0.001
APACHE Ⅱ score	−0.537	<0.001	0.533	<0.001
OI	0.516	<0.001	−0.529	<0.001
RI	−0.527	<0.001	0.504	<0.001

### Predictive value of serum levels of miR-342 and miR-148a for the occurrence of acute lung injury in septic patients

**Figure 3:**
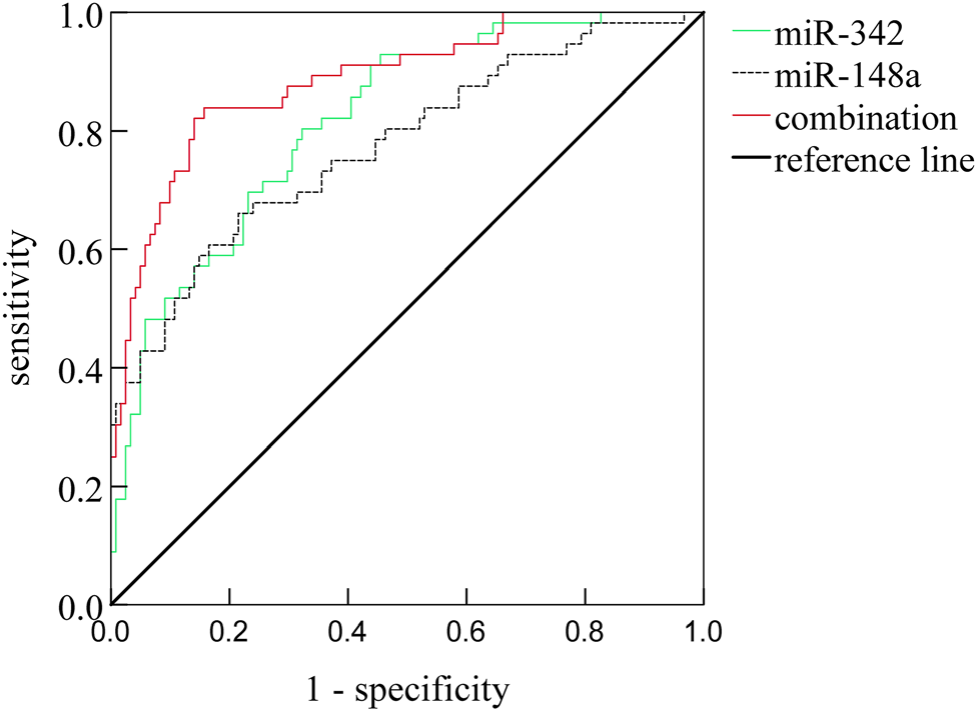
ROC curves of serum levels of miR-342 and miR-148a for the prediction of acute lung injury in septic patients.

Receiver operating characteristic (ROC) curve analysis was conducted to assess the utility of serum miR-342, miR-148a, and their combination in diagnosing ALI among sepsis patients. The area under the ROC curve (AUC) for diagnosing ALI was 0.818 (95 % CI: 0.753–0.882) for miR-342 alone, 0.775 (95 % CI: 0.698–0.853) for miR-148a alone, and 0.896 (95 % CI: 0.832–0.940) for their combination. The combined model demonstrated significantly improved diagnostic performance compared to miR-342 (Z=2.005, p<0.05) and miR-148a (Z=2.551, p<0.05) individually. At optimal cut-off values of 0.88 for miR-342 and 1.20 for miR-148a, the sensitivities were 80.48 and 73.29 %, and specificities were 67.84 and 62.82 %, respectively. For the combined model, the sensitivity was 71.46 % and specificity was 89.34 %. See Figure 3.

### Logistic regression analysis of factors associated with the development of acute lung injury in septic patients

Multivariate logistic regression analysis was performed to identify independent predictors of ALI in septic patients. The dependent variable was ALI status (0=no, 1=yes), and independent variables included serum miR-342, miR-148a, PaCO2, APACHE II score, OI, and RI. Multicollinearity diagnostics indicated no significant collinearity among variables (all tolerance>0.1, all VIF<10) The results showed that miR-342 was a protective factor for the development of acute lung injury in septic patients, while miR-148a was an independent risk factor (p<0.05). See Table 7.

**Table 7:** Logistic regression analysis of factors associated with the development of acute lung injury in septic patients.

Influence factor	B	SE	Waldχ^2^	p-Value	OR	95%CI	Tolerance	VIF
miR-342	−0.123	0.039	9.995	0.002	0.884	0.819–0.954	0.674	2.145
miR-148a	1.080	0.330	10.720	0.001	2.946	1.543–5.625	0.816	2.073
PaCO_2_	0.044	0.132	0.111	0.739	1.045	0.807–1.354	0.805	1.926
APACHE Ⅱ score	0.038	0.107	0.128	0.721	1.039	0.842–1.281	0.834	1.608
OI	0.099	0.113	0.767	0.381	1.104	0.885–1.378	0.868	1.547
RI	0.157	0.148	1.125	0.289	1.170	0.875–1.564	0.710	2.166

## Discussion

Acute lung injury (ALI) is a prevalent pulmonary complication in critically ill patients, frequently triggered by sepsis and characterized by a dysregulated inflammatory response and endothelial dysfunction [15]. The prognosis of acute lung injury patients is poor, with a significant impact on long-term quality of life. Although therapeutic strategies such as conservative fluid management, low tidal volume ventilation, and anti-inflammatory treatments are widely employed, improvements in clinical outcomes for ALI are still urgently needed [16], 17]. Consequently, a deeper understanding of the mechanisms underlying sepsis-induced ALI is crucial for developing novel and more effective therapeutic intervention.

MicroRNAs (miRNAs) are small regulatory RNA molecules with lengths of 19–21 nucleotides that inhibit the translation of target genes by binding to their 3′ untranslated region (UTR) [18]. MiRNAs are involved in the regulation of almost all cellular and physiological processes, including developmental transitions, cell apoptosis, cell cycle, metabolism, host-pathogen interactions, and importantly, regulation of inflammatory responses. They not only directly modulate macrophage polarization and related inflammatory responses but also participate in the inflammatory process by regulating downstream inflammatory factors [19], 20]. MiR-342 has been closely associated with inflammation and infectious diseases, and its regulatory role in inflammation has been confirmed in previous studies. Kim et al. [21]found that the anti-inflammatory effects of glucocorticoids were mediated by Foxp3 (+) regulatory T cells through a miR-342-dependent mechanism. Fu et al. [22] demonstrated that miR-342 controls susceptibility to *Mycobacterium tuberculosis* by regulating inflammation and cell death. A recent study [23] discovered that miR-342 expression is suppressed in the serum of sepsis-associated acute kidney injury patients. Our findings align with this, showing reduced serum miR-342 in septic patients who developed ALI; furthermore, regression analysis identified miR-342 as a protective factor against ALI in this cohort. Moreover, serum miR-342 levels were significantly lower in sepsis patients compared to those with uncomplicated pneumonia, reinforcing its association with the systemic response in sepsis and its potential protective role against ALI development within this group This indicates a close association between miR-342 and sepsis-induced acute lung injury, and compared to ordinary pneumonia patients, sepsis patients may have higher levels of inflammation and a greater degree of abnormal miR-342 expression. Given the established anti-inflammatory roles of miR-342, its downregulation in our study likely contributes to an amplified inflammatory response, thereby exacerbating lung injury in septic patients. Based on the findings of Zhu et al. [9], the decreased level of miR-342 may also promote sepsis-induced acute lung injury by upregulating MAPK1 expression. Additionally, the study by Hayek et al. [24] found that low expression of miR-342-5p can promote damage and senescence of alveolar type II cells, leading to idiopathic pulmonary fibrosis. Therefore, it is speculated that the low expression of miR-342 in this study may also play an important role in sepsis-induced acute lung injury by promoting damage and senescence of alveolar type II cells, while an increased level of miR-342 may exert a corresponding protective effect.

Members of the miR-148a family have been suggested as potential sepsis biomarkers and are recognized for their involvement in the inflammatory response of septic patients [25]. Ma et al. [26] found that the miR-148a-related signaling pathway is involved in the inflammatory and oxidative stress response in sepsis-induced acute kidney injury in rats. Bai et al. [27] demonstrated that extracellular vesicles from adipose tissue-derived stem cells influence the Notch-miR-148a-3p axis to regulate macrophage polarization and alleviate sepsis in mice. In this study, it is speculated that miR-148a may directly modulate macrophage polarization to participate in the progression of sepsis-induced acute lung injury, or it may be involved in the inflammatory process by regulating downstream inflammatory factors. In addition, Kadota et al. [28] found that miR-148a is enriched in extracellular vesicles derived from human bronchial epithelial cells (HBEC EV), and these HBEC EVs can inhibit TGF-β-mediated senescence of lung epithelial cells. It is therefore inferred that elevated expression of miR-148a may also influence lung epithelial cell senescence through HBEC EVs, promoting acute lung injury. This further suggests that increased levels of miR-148a are a risk factor for the development of acute lung injury.

The expression of miR-342 decreased and miR-148a increased with the severity of acute lung injury,and correlation analyses further underscore that dysregulated serum levels of miR-342 and miR-148a are significantly associated with indicators of disease severity and impaired respiratory function in septic patients who develop ALI. Interestingly, while PaCO_2_, APACHE II score, OI, and RI correlated with miRNA levels and ALI status, they did not emerge as independent predictors in the multivariate model, suggesting their association with ALI might be confounded or that the miRNAs reflect a more direct pathological link. The observation of ROC curves in this study showed that miR-342 and miR-148a had AUC values of 0.818 and 0.775, respectively, in predicting acute lung injury in septic patients. When combined, the AUC value reached 0.896, with a specificity of 89.34 %. This indicates that the combination of miR-342 and miR-148a has a higher predictive value for acute lung injury, and as serum biomarkers, their detection methods are simple and easy to perform. Clinically, the identified optimal cut-off values for this miRNA signature could help risk-stratify septic patients, potentially guiding earlier interventions to improve outcomes.

However, since this study is retrospective in nature, it did not include the recording or investigation of relevant inflammatory markers. Future research will further explore the correlation between miR-342, miR-148a, and inflammatory factors. In addition, there is currently a lack of extensive research data confirming that miR-342 and miR-148a levels are specifically associated with acute lung injury. Healthy volunteers were not included as a control group, making it impossible to exclude non-specific changes. In this study, these microRNAs were only used as preliminary evaluation tools, and future studies will aim to further investigate their expression specificity in acute lung injury. Third, no basic experimental validation was conducted in this study, and the potential mechanisms by which miR-342 and miR-148a may be involved in acute lung injury remain unclear.

In conclusion, this study demonstrates that decreased serum miR-342 and increased miR-148a levels are associated with ALI development in sepsis patients. Their combined detection shows robust predictive efficacy, offering potential for early risk assessment. While these miRNAs are implicated in regulating inflammatory responses, further research is essential to elucidate their precise roles in ALI pathogenesis and validate their clinical utility. In the future, samples will be collected from septic patients on days 1, 3, and 7 of the disease course to analyze the dynamic changes of miR-342 and miR-148a during disease progression or recovery.
